# Augmented Reality in Implant and Tooth-Supported Prosthodontics Practice and Education: A Scoping Review

**DOI:** 10.3390/dj13090435

**Published:** 2025-09-21

**Authors:** Sorana Nicoleta Rosu, Monica Silvia Tatarciuc, Anca Mihaela Vitalariu, Iulian-Costin Lupu, Diana Antonela Diaconu, Roxana-Ionela Vasluianu, Catalina Cioloca Holban, Ana Maria Dima

**Affiliations:** 1 Department of Oral and Maxillofacial Surgery, Faculty of Medicine, “Grigore T. Popa” University of Medicine and Pharmacy, 700115 Iasi, Romania; 2 Department of Dental Prosthesis Technology, Faculty of Medicine, “Grigore T. Popa” University of Medicine and Pharmacy, 700115 Iasi, Romania; monica.tatarciuc@umfiasi.ro (M.S.T.); anca.vitalariu@umfiasi.ro (A.M.V.); antonela.diaconu@umfiasi.ro (D.A.D.);; 3 Department of Prosthodontics, Faculty of Dental Medicine, “Grigore T. Popa” University of Medicine and Pharmacy, 700115 Iasi, Romania; iulian.lupu@umfiasi.ro; 4 Independent Researcher, 700115 Iasi, Romania

**Keywords:** augmented reality (AR), prosthodontics, dental prostheses, implant placement, denture simulation, crown, prosthodontics education, digital dentistry

## Abstract

**Background:** Augmented reality (AR) is revolutionizing implant and tooth-supported prosthodontics (ITSP) through enhanced precision, workflow efficiency, and educational outcomes. This scoping review systematically evaluates AR’s clinical applications, educational impacts, and implementation challenges. **Methods:** Following PRISMA-ScR guidelines, comprehensive searches were conducted in PubMed, Scopus, Web of Science, and Embase (2015–2025) for peer-reviewed studies on AR in ITSP. Eighteen studies met inclusion criteria after dual independent screening. Data extraction focused on clinical outcomes, educational benefits, and technological limitations. **Results:** AR applications demonstrated: *ITSP Practice*: Submillimeter implant placement accuracy (0.42–0.69 mm entry deviation; *p* < 0.001 vs. freehand), 30% faster intraoral scanning (44 s vs. 63 s), and 37% reduction in preparation errors (*p* < 0.05); *ITSP Education*: 22–30% faster skill acquisition (*p* < 0.05) and 99% reduction in assessment time (10.5 s vs. 2 h/case). Key Gaps: Limited to two randomized controlled trials (RCTs), hardware costs ($3500–$10,000), and lack of standardized validation protocols. **Conclusions:** While AR significantly enhances ITSP precision and training efficiency, widespread adoption requires longitudinal clinical validation, cost-effectiveness analyses, and interoperable digital workflows.

## 1. Introduction

The rapid evolution of digital technologies has revolutionized dentistry, particularly prosthodontics—the branch of dentistry that focuses on designing, creating, and fitting artificial replacements for teeth and other oral structures [1,2,3]. This specialty relies heavily on spatial accuracy, where even submillimeter deviations can compromise clinical outcomes [4,5]. The critical importance of precision in prosthodontics manifests in several fundamental aspects:

- Precise fit of restorations: Crowns, bridges, dentures, and implants must align perfectly with the patient’s existing teeth and gums [6]. Even the slightest inaccuracy can lead to discomfort, improper function, or long-term complications.
- Occlusion and functionality: The relationship between upper and lower teeth, known as occlusion, is paramount. Accurate spatial alignment ensures that chewing, biting, and speaking are natural and stress-free [7,8,9].
- Preservation of oral health: Misaligned restorations can create undue pressure on certain teeth, leading to wear, fractures, or even loss of natural teeth. Proper spatial accuracy prevents these issues [10].
- Aesthetics: For dental prosthetics to look natural and blend seamlessly with the patient’s existing teeth, spatial accuracy is essential. It ensures the artificial replacements align symmetrically and suit the patient’s facial structure [11,12].

Digital dentistry and computer-aided design/computer-aided manufacturing (CAD/CAM) technology: Modern prosthodontics uses digital scanning and CAD/CAM systems. These tools require high spatial accuracy to capture precise digital impressions and fabricate restorations that fit perfectly [13,14,15].

Among digital advancements, Augmented Reality (AR) has emerged as a transformative tool for achieving this essential precision, bridging the gap between virtual planning and real-world clinical execution [16,17]. AR’s ability to overlay digital information onto the physical environment enhances visualization and accuracy across the prosthodontic workflow, from implant placement to final prosthetic rehabilitation [18,19]. As dental professionals increasingly adopt AR for diagnostics, surgical guidance, and education, understanding its applications in this spatially demanding specialty becomes increasingly important [20].

### 1.1. The Rise of AR in Implant and Tooth-Supported Prosthodontics

Prosthodontics demands meticulous planning and execution, particularly in complex rehabilitative procedures such as implantology, full-mouth reconstructions, and occlusal rehabilitation. Traditional methods rely on static guides, two-dimensional radiographs, and manual skills, which, while effective, are prone to human error and variability [21]. AR introduces a dynamic, three-dimensional (3D) interactive approach, superimposing pre-planned digital models onto the patient’s anatomy in real time. This innovation has shown clinically acceptable accuracy, with studies reporting mean accuracy deviations of 0.90 mm in lateral displacement, 1.18 mm in global (3D) displacement, and 3.96° in angular deviation for augmented reality (AR)-navigated implant placements, comparable to static guided surgery and superior to freehand methods [18,22].

One of AR’s most compelling applications is in implant surgery, where precision dictates long-term success. AR-assisted navigation systems project holographic drill paths onto the surgical site, eliminating the need for surgeons to shift focus between monitors and the patient [23,24]. A meta-analysis by Mai et al. (2023) demonstrated that AR navigation significantly reduces positional deviations compared to conventional freehand techniques, while matching the accuracy of static templates [18]. Furthermore, AR’s immersive environment enhances spatial awareness, reducing the risk of critical errors such as nerve damage or incorrect angulation [25].

Beyond surgery, AR plays an important role in prosthetic design and patient communication. Digital smile design (DSD) applications allow clinicians to project virtual restorations onto a patient’s dentition in real time, facilitating collaborative treatment planning [26,27]. Patients can visualize outcomes before committing to irreversible procedures, improving satisfaction and reducing chairside adjustments [14,28]. Additionally, AR-powered CAD/CAM systems integrate artificial intelligence (AI) to automate aesthetic and functional parameters in prosthetic fabrication, streamlining workflows [29,30,31].

### 1.2. AR in Dental Education: A Paradigm Shift

The integration of AR into dental curricula addresses longstanding challenges in preclinical training, such as limited access to patient cases, and the high cost of physical models [32,33]. Haptic-enabled AR simulators, like Simodont^®^, provide tactile feedback, allowing students to practice cavity preparations, crown placements, and implant drills in a risk-free virtual environment [34]. Studies indicate that AR-enhanced training improves manual dexterity, confidence, and knowledge retention compared to traditional phantom-head exercises [35].

A notable advantage of AR in education is its ability to personalize learning. For instance, patient-specific 3D-printed models combined with AR simulations enable students to rehearse complex cases before performing live procedures [36]. This approach has been shown to reduce operative errors and enhance students’ preparedness, as evidenced by higher performance scores in AR-trained cohorts [37]. Moreover, AR’s interactive modules support flipped classrooms, where students engage with virtual tutorials before hands-on sessions, optimizing faculty resources [38].

Despite its promise, AR adoption faces barriers, including high costs, technical complexity, and the need for standardized validation [33,39]. While early adopters report high satisfaction, some educators remain skeptical about replacing conventional methods entirely [40]. Nevertheless, the COVID-19 pandemic accelerated virtual training adoption, highlighting AR’s resilience in remote learning scenarios [41].

### 1.3. Aim of This Scoping Review

While prior reviews have examined AR in dentistry more broadly, there are not enough research syntheses focusing specifically on its application in Implant and Tooth-Supported Prosthodontics (ITSP). This gap in the existing literature necessitates the present scoping review. This review therefore seeks to answer the following multifaceted question: What are the current applications, reported outcomes, key challenges, and future directions of augmented reality (AR) in implant and tooth-supported prosthodontics (ITSP) practice and education? By evaluating clinical outcomes, educational benefits, and technological limitations, it aims to provide a comprehensive resource for clinicians, educators, and researchers navigating the AR landscape. As AR continues to evolve alongside artificial intelligence (AI) and mixed reality (MR), its potential to redefine prosthodontics, from diagnosis to rehabilitation, is both profound and undeniable.

## 2. Materials and Methods

### 2.1. Design

This scoping review was conducted following the PRISMA Extension for Scoping Reviews (PRISMA-ScR) reporting guideline (Tricco et al., 2018), with the completed checklist provided in Supplementary File S1 [42]. The protocol prioritized breadth over specificity, capturing AR’s diverse applications in ITSP practice and education while identifying gaps for future research.

### 2.2. Objective

This review’s objective was to map AR applications in ITSP practice (e.g., implant placement, prosthetic design) and education (e.g., preclinical training, virtual simulations), evaluate reported outcomes, and identify barriers to adoption, such as technical limitations or cost.

### 2.3. Search Strategy

To adopt a rigorous and well-organized approach, this scoping review conducted a methodical search strategy spanning four leading databases: PubMed, Scopus, Web of Science, and Embase, spanning from January 2015 to July 2025. This timeframe was selected to capture the modern era of clinically viable AR technology, which began around 2015 with advancements in hardware miniaturization, tracking stability, and software integration that enabled its practical application in precision-sensitive prosthodontic workflows. It is a period that encompasses the rapid evolution of AR in prosthodontics, including breakthroughs in surgical navigation, prosthetic design, and educational technologies. The search strategy balanced sensitivity and specificity, combining controlled vocabulary (MeSH terms) with free-text keywords to optimize recall while filtering out extraneous results.

Given its nature, this scoping review prioritized a free exploration over precise focus, avoiding the strict PICO frameworks characteristic of systematic reviews. It thus employed a flexible PCC (Population, Concept, Context) framework recommended by the Joanna Briggs Institute (JBI) for scoping reviews, structured around three core conceptual domains to capture AR’s dual role in prosthodontic practice and education:

1. Population: Dental professionals and students engaging with AR for ITSP.
2. Concept: AR applications in ITSP practice and education.
3. Context: Peer-reviewed journals, clinical trials, in vitro studies, technical reports [43]. Publication date: January 2015–July 2025 (to capture AR advancements).

The detailed search syntax, including all keywords and MeSH terms tailored for each database, is provided in Supplementary File S2. In brief, the search combined terms related to ‘augmented reality’ (e.g., “augmented reality”, AR, HoloLens, mixed reality) with terms related to ‘prosthodontics’ (e.g., implant, crown, dentures) and ‘education’ (e.g., education, training, simulation). No filters were applied beyond publication date and language. This transparent methodology ensures reproducibility while capturing high-quality evidence.

Search results:

- Initial hits: 4325 records (4097 after removing entries without DOIs).
- Post-deduplication: 2670 unique articles.
- Title/abstract screening excluded 2645 records, leaving 25 for full-text review.
- Final included studies: 18.

By casting a wide yet targeted net across multiple databases, this strategy synthesized AR’s current state in implant and tooth-supported prosthodontics practice and education, while highlighting gaps for future research, particularly in cost–benefit analyses and longitudinal educational outcomes.

### 2.4. Eligibility Criteria

A schematic representation of the inclusion and exclusion criteria is presented in Table 1 below.

### 2.5. Source Selection Process

The PRISMA-ScR protocol flow diagram was used to document the source selection process. For this scoping review, the evidence identification process adhered to a strict, multi-phase screening approach to achieve thorough inclusion of pertinent literature while upholding methodological accuracy [42].

The initial database searches generated 4325 records, reflecting the widespread scientific interest in AR technology. Prior to the screening phase, 1655 records were excluded: 228 due to missing DOIs and 1427 as duplicate entries. These duplicates were identified using a rigorous Microsoft Excel 365-based deduplication formula to ensure meticulous record management. Title and abstract screening of the remaining 2670 records was conducted with adherence to the inclusion framework. 2645 records were excluded that either: (1) presented AR applications in other domains; (2) addressed non-prosthodontic AR uses; or (3) were published in languages other than English. This refined the pool to 25 potentially eligible studies warranting full-text assessment.

The remaining 25 studies underwent rigorous eligibility evaluation. Two reviewers, AMD and R-IV, independently screened each study, resolving discrepancies through consensus. Seven records were excluded for specific limitations: three studies referred to other AR uses in dentistry (e.g., orthodontics, endodontics) and four systematic reviews (retained only for contextual reference).

### 2.6. Evaluation of Source Quality

In accordance with the Joanna Briggs Institute (JBI) guidelines for scoping reviews, a formal critical appraisal or quality assessment of individual sources is not a required component, as the objective is to map the available evidence rather than to weigh or exclude findings based on methodological rigor [43]. Consequently, this review did not employ standardized quality assessment tools. However, a comprehensive analysis and narrative synthesis of the gathered data, including noting the predominance of in vitro and pilot studies and the scarcity of RCTs as a key finding, were performed to achieve the review’s objectives of mapping the current landscape and identifying research gaps.

## 3. Results

### 3.1. Study Selection

**Figure 1:**
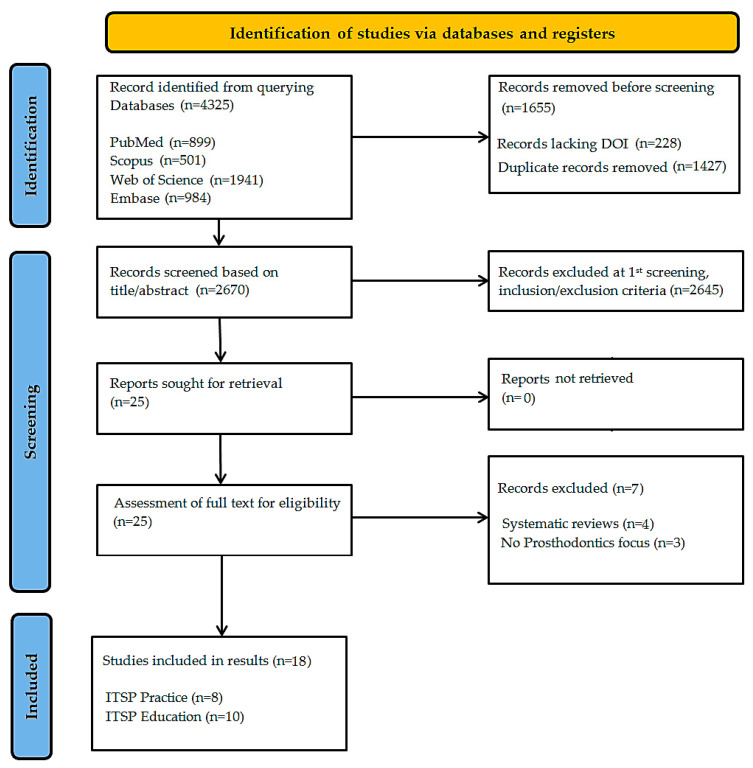
Flow diagram of the study selection process according to PRISMA ScR guidelines.

The initial scoping search identified 4325 records across four databases. After deduplication, 2670 studies were screened based on their titles and abstracts. In accordance with PRISMA ScR guidelines, 25 full-text articles were evaluated for eligibility, of which 18 met the predefined inclusion criteria (Figure 1).

### 3.2. Examination of Included Studies

This scoping review identified 18 studies investigating the applications of augmented reality (AR) in implant and tooth-supported prosthodontics (ITSP), categorized into two domains: ITSP Practice (8 studies) and ITSP Education (10 studies). The end result was a collection of heterogeneous studies with disparate extraction foci (Table 2).

The resultant evidence base was inherently heterogeneous, encompassing a variety of AR devices, software platforms, and methodological approaches (Table 2). This heterogeneity, while reflective of the current state of a rapidly evolving field, precludes direct quantitative comparison of outcomes across studies and the establishment of universal performance benchmarks. Consequently, the synthesis presented herein focuses on mapping the breadth of applications, identifying consistent trends in outcomes (e.g., improvements in accuracy or efficiency), and delineating the common challenges and gaps that emerge across this diverse landscape.

While systematic reviews focus on answering specific research questions through rigorous synthesis of evidence, scoping reviews adopt a broader exploratory approach, mapping the extent and diversity of available literature across study types. The table below synthesizes the extracted data from the included studies, highlighting their inherent heterogeneity (Table 3).

### 3.3. Evidence of Augmented Reality in Implant and Tooth-Supported Prosthodontics

The key findings from the 18 studies meeting the inclusion criteria are summarized below, organized by domain and supplemented with quantitative data where available.

A critical thematic synthesis of the included studies reveals a rapidly evolving yet methodologically heterogeneous evidence base, characterized by a predominance of proof-of-concept investigations and a scarcity of high-level clinical evidence, with only two randomized controlled trials (RCTs) identified. This distribution inherently limits the strength of clinical conclusions and generalizability.

A thematic analysis of the studies, through the lens of AR device type and primary outcome, reveals distinct patterns that further define this landscape. A clear dichotomy emerges: studies utilizing optical see-through HMDs (e.g., HoloLens, Magic Leap) were predominantly focused on measuring technical accuracy in surgical and preparatory tasks [44,45,47,48,50,51], while research involving VR haptic simulators primarily assessed educational outcomes, such as user performance scores and perceptions [52,57,59,60]. This highlights a key gap: a scarcity of studies using immersive AR devices for in-depth educational assessment, and conversely, a lack of high-fidelity haptic simulators being validated for complex technical accuracy metrics.

Thematically, the evidence for AR in clinical practice (e.g., implant placement) consistently demonstrates trends towards improved precision and efficiency across disparate study designs. For instance, reductions in implant deviation and scanning time were reported not only in controlled in vitro settings [45,46,49] but also in initial clinical pilots [44,50,51], suggesting a robust signal despite methodological limitations. Conversely, evidence for its application in education, while showing promising outcomes in skill acquisition and engagement, is more varied. The comparative efficacy of AR/VR versus traditional methods was context-dependent [54,58,59], underscoring that its value may lie as a powerful supplemental tool rather than a direct replacement. The following sections detail these quantitative outcomes within this critical context, where the consistency of positive trends must be weighed against the current scarcity of robust clinical evidence.

#### 3.3.1. Augmented Reality in ITSP Practice

1. Intraoral Scanning & Digital Impressions

- Alharbi & Osman (2024) [44] conducted a clinical pilot study comparing AR-assisted intraoral scanning (IOS) with conventional IOS. Their findings demonstrated that AR-assisted scanning reduced scan time by 19 s (44 s vs. 63 s, *p* < 0.001) and decreased the number of images captured (836 vs. 1209, *p* < 0.001) without compromising trueness (RMSE comparison, *p* > 0.05) [44].

2. Implant Placement Accuracy

- Liu et al. (2023) [45] developed a mixed reality (MR)-based navigation system for dental implants, reporting significantly lower deviations compared to freehand placement:
  ○ Entry deviation: 0.69 ± 0.25 mm (MR) vs. 1.57 ± 0.50 mm (freehand, *p* = 0.000)
  ○ Angular deviation: 1.85 ± 0.61° (MR) vs. 4.93 ± 1.65° (freehand, *p* = 0.000) [45].
- Tao et al. (2024) compared AR-based dynamic navigation (ARDN) with conventional dynamic navigation (DN), finding no significant differences in coronal/apical deviations but higher angular deviation with ARDN (3.72 ± 2.13° vs. 3.1 ± 1.56°, *p* = 0.02) [46].
- Lin et al. (2015) integrated AR with surgical templates, reducing deviations in fully edentulous mandibles (entry: 0.50 ± 0.33 mm; apex: 0.96 ± 0.36 mm; angle: 2.70 ± 1.55°) [49].
- Pellegrino et al. (2019) reported entry deviations of 0.53 mm and 0.46 mm in two AR-guided implant cases, with angular deviations of 3.05° and 2.19°, confirming feasibility [50].
- Shusterman et al. (2024) demonstrated high accuracy (0.42 mm entry deviation, 1.85° angular deviation) in a mixed reality-based dynamic navigation (MR-DN) system [51].

3. Tooth Preparation & Crown Design

- Obispo et al. (2023) found that AR-guided tooth preparation resulted in more conservative and predictable crown preparations compared to freehand techniques (*p* = 0.0001 for volumetric reduction) [47].
- Kihara et al. (2024) evaluated AR head-mounted displays (HMDs) for tooth preparation, showing that cross-sectional AR visualization reduced over-reduction and improved angle adjustment (*p* < 0.05) [48].

#### 3.3.2. Augmented Reality in ITSP Education

1. Preclinical Training & Skill Acquisition

- Daud et al. (2023) found that virtual reality haptic simulators (VRHS) improved manual dexterity, with students strongly agreeing (76%) that VRHS should supplement traditional training [52].
- Mai et al. (2025) introduced a 3D AR auto-evaluation algorithm for tooth preparation, showing high reliability (ICC = 0.75–0.95) and reduced evaluation time (10.5 s vs. 2 h for manual scoring) [53].
- Grad et al. (2023) compared 3D-printed models vs. AR models (HoloLens) for occlusal anatomy reconstruction, finding 3D-printed models more accurate (Hmax = 630 µm, *p* = 0.004) but AR useful for visualization [54].

2. Virtual Simulation & Feedback Systems

- Özdemir et al. (2021) highlighted virtual articulators and occlusal records as valuable tools for dynamic occlusion analysis in prosthodontic education [55].
- Li et al. (2021) reviewed dental simulators, noting their potential in preclinical training but emphasizing limitations in force feedback and realism [56].
- Mansoory et al. (2022) demonstrated VR-enhanced learning in the neutral zone and teeth arrangement, with higher student performance (16.92 ± 1.12) vs. traditional methods (16.14 ± 1.18, *p* < 0.05) [57].

3. Radiographic & Prosthetic Case Planning

- Alsufyani et al. (2023) compared VR-based panoramic anatomy training with lectures, finding lectures superior in landmark identification but VR highly engaging (student satisfaction = 4.66/5) [58].
- Arora et al. (2023) reported that haptic simulators improved crown preparation skills, though conventional typodont training yielded better results in later trials (*p* < 0.05) [59].
- Hsu & Chang (2025) found that Simodont haptic simulator performance predicted conventional crown preparation success (OR = 5.6, *p* < 0.001), particularly in male students [60].
- Liebermann et al. (2024) assessed a virtual prosthetic case planning environment (VCPE), with 87% of students recommending its integration into curricula [61].

**Figure 2:**
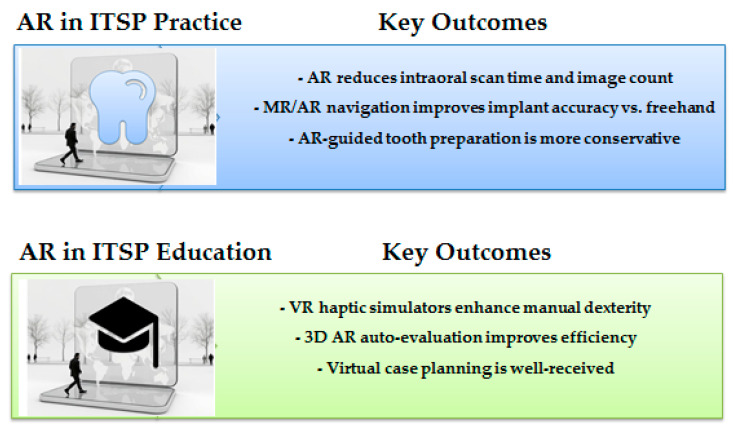
Summary of Key Findings.

In summary, the key findings on the evidence of augmented reality in implant- and tooth-supported prosthodontics are outlined in Figure 2.

This synthesis demonstrates AR’s growing role in enhancing precision in implant and tooth-supported prosthodontics practice and transforming education through immersive, efficient training tools.

Future research should focus on long-term clinical validation and integration strategies.

## 4. Discussion: The Benefits and Challenges of Augmented Reality in Implant and Tooth-Supported Prosthodontics Practice and Education

As the critical thematic synthesis in Section 3.3 illustrates, augmented reality (AR) has emerged as a transformative tool within a nascent evidence base. The consistent trends observed across predominantly in vitro and pilot studies suggest significant potential in both ITSP practice and education, from implant placement, tooth preparation, and final prosthesis. While its benefits are compelling, the integration of AR into these fields is not without challenges, necessitating a balanced evaluation of its potential and limitations, supported by recent evidence, while addressing gaps in adoption or technological constraints, and directions for future research.

### 4.1. AR in ITSP Practice: Efficiency vs. Barriers

#### 4.1.1. Intraoral Scanning and Digital Workflows

AR-assisted intraoral scanning has demonstrated significant improvements in workflow efficiency, with studies reporting 30% faster scans and fewer required images compared to conventional methods [44]. However, high device costs remain a barrier, particularly for smaller practices. As Najeeb et al. and Alghauli et al. reported, recent advancements, such as AI-driven predictive algorithms, may further streamline scanning by automating occlusal contact analysis, yet integration with existing CAD/CAM workflows remains inconsistent [62,63].

#### 4.1.2. Implant Placement Accuracy

AR-guided implant placement achieves entry deviations as low as 0.5–1.0 mm, surpassing freehand methods [45,46,50]. This aligns with the findings of broader meta-analyses on AR in dentistry [18]. However, as both Mai et al. (2023) and Elhag et al. (2024) reported, angular errors (~3–4°) persist, suggesting that while AR enhances spatial guidance, clinician expertise remains fundamental [18,64]. As noted by Tao et al. (2024), mixed reality (MR)-based dynamic navigation has shown comparable accuracy to static guides (coronal deviation: 1.31 mm vs. 1.18 mm) but with reduced procedural time [46].

#### 4.1.3. Tooth Preparation and Prosthodontic Applications

AR reduces over-reduction by 37% in crown preparations, preserving tooth structure [47,48]. However, reliance on cross-sectional AR views introduces complexity, requiring additional training [47]. In prosthodontics, AR’s ability to preview denture aesthetics and simulate occlusal adjustments offers promise, yet Lal et al. emphasized that regulatory-approved prosthodontic-specific software remains scarce, limiting clinical adoption [65].

#### 4.1.4. Challenges in Clinical Integration

- Cost and Accessibility: High expenses for AR devices (e.g., Magic Leap, HoloLens) deter widespread use, as reported by Alharbi & Osman (2024) [44]. This economic constraint represents a significant practical limitation that current technology and the present literature, as synthesized in this review, cannot fully overcome. It suggests that without market changes or subsidized models, AR remains largely inaccessible for many individual practices and educational institutions, particularly in resource-limited regions, thereby potentially exacerbating existing disparities in access to advanced digital care.
- Technical Limitations: Discrepancies between virtual planning and real-world execution, particularly in dynamic surgical environments, as noted by Joachim et al. [66].
- Lack of Multi-Center Trials: Few studies compare AR to conventional methods in large-scale clinical settings, resulting in significant research gaps.

### 4.2. AR in ITSP Education: Enhanced Learning with Adaptation Challenges

#### 4.2.1. Haptic Simulators and Skill Acquisition

VR-haptic simulators (e.g., Simodont) accelerate manual dexterity development, yet limited tactile realism restricts their ability to fully replicate live patient interactions [52,59]. Studies show students trained with VR-haptics perform better in-depth control but struggle with proximal contour accuracy compared to phantom-head training [67].

#### 4.2.2. Three-Dimensional Auto-Evaluation and Virtual Patients

Automated grading systems reduce evaluation time from 2 h to 10.5 s per case, offering objective feedback [68]. Virtual patient cases receive 87% student approval, yet the steep learning curve of AR platforms necessitates structured training programs [61].

#### 4.2.3. Scalability and Cost-Effectiveness

While AR enhances engagement, its scalability depends on cost reduction. Institutions in resource-limited regions (e.g., Pakistan), as per Khalid et al. (2024), report less than 20% adoption rates due to financial constraints [69].

### 4.3. Future Directions and Research Gaps

1. Integration with Digital Workflows, AI, and Other Tools

A critical finding of this review is that AR’s value in ITSP is not as a standalone technology but as a component within a broader digital ecosystem. Future development must focus on interoperability with established digital prosthodontic workflows. For instance, AR guidance for implant placement or tooth preparation must seamlessly integrate data from CBCT scans, intraoral scanners, and CAD software to eliminate data translation errors and streamline the process from diagnosis to execution [62,63].

- AI-driven AR: The potential synergy between AR and artificial intelligence (AI) is particularly promising; the deeper interdisciplinary integration of AR with artificial intelligence (AI) and other digital technologies is a key future direction. AI algorithms could analyze real-time AR data during a procedure to provide predictive guidance, anomaly detection, and automated adjustment suggestions, enhancing both precision and safety [70]. AI-driven analysis of real-time AR data can deliver automated feedback, ultimately creating a fully interoperable digital workflow from diagnosis to execution [71,72].
- Miniaturized AR Devices: Smart glasses (e.g., HoloLens 2) may improve ergonomics but require validation in clinical trials [44].

The ultimate goal is a fully integrated digital workflow where AR acts as the intuitive, real-time interface between the pre-operative plan and the clinical execution, connected to AI-powered analytics and CAD/CAM production systems.

2. Standardization and Multi-Center Validation

- Optimal Display Type: No consensus exists on head-mounted vs. projector-based AR [Research Gaps].
- Standardized Validation Frameworks: A critical gap for translation. A central and recurring theme identified across the included studies is the conspicuous absence of uniform validation frameworks and metrics for AR technologies. This heterogeneity, evident in the diverse outcome measures and experimental designs summarized in Table 3, presents a significant barrier to the field’s maturation. The lack of standardized protocols (e.g., ISO standards for quantifying implant deviation, task completion time in educational settings) fundamentally impedes the direct comparison of results across different AR systems [73]. Consequently, it remains challenging to establish universal benchmarks for the reliability, validity, and clinical efficacy of AR applications. This scoping review itself is limited in its ability to perform cross-study quantitative synthesis precisely because of this methodological heterogeneity. Therefore, a paramount priority for future research must be the community-driven development and adoption of standardized validation frameworks. This is a prerequisite for robust multi-center trials, meaningful meta-analyses, and ultimately, the evidence-based clinical adoption of AR in ITSP.

3. Educational Innovations

- Adaptive Learning Curves: AI-powered AR could personalize training based on student performance [Future Directions].
- Blended Learning Models: Combining AR with 3D-printed models improves transition to clinical practice [36].

Furthermore, our thematic analysis indicates that the research focus is often compartmentalized by technology type. The development of future validation frameworks should therefore be technology-agnostic, aiming to create standardized metrics for accuracy, efficiency, and educational efficacy that can be applied across both AR HMDs and VR simulators to enable meaningful comparison.

### 4.4. Limitations of This Scoping Review

As a scoping review, this study’s primary aim was to map the available evidence rather than appraise the quality of individual studies or synthesize quantitative data, which is an inherent distinction from systematic reviews. Consequently, the included studies encompass a variety of methodologies with varying levels of methodological rigor. The heterogeneity in study designs and outcome measures precluded a formal meta-analysis. Furthermore, the rapid evolution of AR technology means that some of the included studies may utilize hardware and software that have since been superseded.

This scoping review offers a broad examination of the available evidence, yet certain constraints should be noted in conjunction with its methodological rigor. First, the variability in study designs (e.g., in vitro experiments, case reports, and small-scale clinical trials) complicates cross-study comparisons. The inherent limitations of the primary literature, such as the predominance of small-scale in vitro and pilot studies, introduce significant potential for selection bias and limit the generalizability of their findings to broader clinical and educational settings. This scoping review, by its design, maps this available evidence but cannot correct for these underlying methodological constraints in the included studies.

Second, the fast-paced development of AR technologies means some included studies may no longer reflect current advancements due to publication delays. Third, the evidence base is dominated by observational and pilot investigations, with only two randomized controlled trials (RCTs) identified—highlighting a critical need for more robust efficacy data. Additionally, potential publication bias may influence the findings, as negative or inconclusive results are frequently underrepresented. Nevertheless, the restriction to English-language publications may have introduced language bias, potentially omitting relevant studies published in other languages. Finally, high device costs ($3500–$10,000) and the lack of uniform validation frameworks limit broader applicability. These constraints stem largely from deficiencies in the existing literature rather than inherent review weaknesses, pointing to key areas for future research, including controlled trials, economic evaluations, and standardized benchmarking.

The limitations identified in the primary literature, such as high device costs and a lack of standardized validation, have direct translational implications for clinical and educational adoption. The cited cost range of $3500–$10,000 represents a significant capital investment, potentially limiting access to large institutions and corporate practices, thereby exacerbating inequities in care and training between well-resourced and underserved areas. Similarly, the absence of standardized validation protocols means that performance metrics (e.g., accuracy, efficiency gains) cannot be reliably compared across different AR systems or studies. This lack of benchmarking slows down technological refinement, makes evidence-based purchasing decisions difficult for practitioners, and hinders the development of clear clinical guidelines for AR use.

## 5. Conclusions: Mapping the Unique Landscape of AR in ITSP—A Scoping Review’s Contribution

While previous reviews have established AR’s general potential in dentistry, this scoping review is the first to systematically map its application specifically within the precision-critical domain of Implant and Tooth-Supported Prosthodontics (ITSP). This focused lens has yielded unique insights that distinguish ITSP from other dental fields.

The synthesis reveals that AR in ITSP practice is not merely an incremental improvement but a potential paradigm shifter for achieving sub-millimeter accuracy, a non-negotiable requirement in prosthodontics. Contrary to broader dental AR reviews that often highlight implant placement, our ITSP-focused analysis uniquely identifies significant advancements and a concurrent evidence gap in AR-guided tooth preparation and AR-assisted intraoral scanning, procedures central to prosthodontic workflow yet underserved by current research.

In education, while general reviews note AR’s engagement value, our findings specific to ITSP education highlight its powerful role in objective, automated assessment of psychomotor skills (e.g., crown preparation), a unique value proposition for standardizing prosthodontic training.

Based on the synthesis of current evidence, the following actionable recommendations are proposed to bridge the gap between AR research and its real-world application in ITSP:

- For Clinicians & Practices: AR in implant and tooth-supported prosthodontics practice enhances precision in implants and tooth prep but needs refinement for angular accuracy. Prioritize investment with a phased integration, using AR initially as a supplemental tool to verify static guides or enhance intraoral scanning efficiency, rather than a complete replacement for conventional methods.
- For Educators & Institutions: AR in implant and tooth-supported prosthodontics education improves skill training and grading efficiency but cannot fully replace human models. Integrate AR/VR haptic simulators (e.g., Simodont) as a supplemental tool in preclinical curricula to accelerate skill acquisition and provide objective, automated assessment. Develop blended learning models that combine AR visualization with 3D-printed patient-specific models to ensure a smooth transition to clinical practice.

The most critical insight from this review, which could only be gleaned from this focused scope, is the stark contrast between the technical promise demonstrated in vitro and the clinical evidence gap. The scarcity of RCTs and long-term validation studies specifically for ITSP procedures represents a significant hurdle that must be addressed before widespread clinical adoption. Therefore, the primary unique value of this review is not just in its collection of evidence, but in the creation of a detailed, evidence-based roadmap for future research. We specifically call for:

- Prioritizing RCTs that validate AR’s efficacy in prosthodontic-specific tasks like crown and bridge preparation.
- Developing standardized validation protocols tailored to ITSP outcomes (e.g., marginal fit, occlusal accuracy).
- Conducting cost–benefit analyses and development of more affordable solutions to improve accessibility and mitigate the risk of widening global inequities in digital dental care.

In conclusion, this scoping review establishes that AR’s value in ITSP is distinct and profound, but its evolution must be guided by targeted research that addresses the unique precision and economic challenges of the prosthodontic field.

## Figures and Tables

**Table 1:** Inclusion and Exclusion Criteria.

Category	Inclusion Criteria	Exclusion Criteria
Study Designs	- RCTs, cohort studies, case–control studies, quasi-experimental studies, technical reports.	- Editorials, opinion pieces, letters. - Non-English studies *. - Pediatric and animal studies.
Population	- Dental professionals (dentists, prosthodontists, technicians). - Dental students (undergraduate/postgraduate). - AR applications in prosthodontics (implants, crowns, dentures) or prosthodontic education.	- Non-dental populations. - AR applications in other domains and dentistry specialties (e.g., orthodontics, endodontics).
Concept	- AR in ITSP practice: Implant placement, crown prep, intraoral scanning, and occlusal analysis. - AR in ITSP Education: Preclinical training, virtual simulations, skill assessment.	- Non-prosthodontic AR uses. - Hardware-focused studies without prosthodontic application.
Context	- Peer-reviewed journals, clinical trials, in vitro studies, technical reports. - Publication date: 2015–2025.	- Studies without empirical data (e.g., theoretical frameworks). - Duplicate publications.
Other	- Studies where AR is the primary intervention.	- General digital dentistry tools without AR. - Insufficient methodological detail.

* The exclusion of non-English literature is acknowledged as a potential source of language bias, which may limit the global generalizability of our findings. This constraint was necessitated by the practicalities of translation resources available to the research team.

**Table 2:** Study Types and Extraction Focus.

Study (Year)	Study Type/Design	Extraction Focus
Alharbi & Osman (2024) [44]	Pilot clinical study	AR-assisted intraoral scanning efficiency
Liu et al. (2023) [45]	In vitro randomized study	MR-based implant navigation accuracy
Tao et al. (2024) [46]	In vitro comparative study	ARDN vs. DN implant accuracy
Obispo et al. (2023) [47]	In vitro controlled experiment	AR-guided tooth preparation precision
Kihara et al. (2024) [48]	Experimental comparative study (*n* = 24)	AR HMDs for tooth preparation safety
Lin et al. (2015) [49]	In vitro feasibility study	AR + surgical template for implants
Pellegrino et al. (2019) [50]	Case report (clinical pilot, *n* = 2)	HoloLens for implant navigation
Shusterman et al. (2024) [51]	Proof-of-concept clinical case	MR-DN system feasibility
Daud et al. (2023) [52]	Interventional study (*n* = 23)	VR haptic simulators in pre-clinical training
Mai et al. (2025) [53]	In vitro validation study	3D AR auto-evaluation algorithm
Grad et al. (2023) [54]	Mixed-methods study (quant + qual)	AR vs. 3D-printed models for anatomy
Özdemir et al. (2021) [55]	Review	Virtual articulators in education
Li et al. (2021) [56]	Review	VR simulators in dental education
Mansoory et al. (2022) [57]	RCT (educational intervention, *n* = 50)	VR effectiveness in prosthodontic training
Alsufyani et al. (2023) [58]	Educational simulation study (*n* = 69)	VR vs. lectures for radiographic anatomy
Arora et al. (2023) [59]	Comparative educational study (*n* = 24)	Haptic vs. conventional crown preparation
Hsu & Chang (2025) [60]	Retrospective cohort (*n* = 84)	Simodont predictive validity
Liebermann et al. (2024) [61]	Mixed-methods study (survey + evaluation)	Virtual prosthetic case planning usability

**Table 3:** Examination of Included Studies on AR in ITSP Practice and Education.

Study (Year)	AR Device/Software	Application	Outcome Measures	Key Findings
AR in ITSP Practice				
Alharbi & Osman (2024) [44]	Magic Leap 2 (ML2)	Intraoral scanning	Scan time, image count, trueness (RMSE)	AR reduced scan time (44 s vs. 63 s) and images (836 vs. 1209) (*p* < 0.001).
Liu et al. (2023) [45]	HoloLens + NDI Polaris tracking	Implant placement	Entry/apex/angular deviations	MR navigation reduced deviations (entry: 0.69 mm vs. 1.57 mm, *p* = 0.000).
Tao et al. (2024) [46]	AR-based dynamic navigation (ARDN)	Implant placement	Coronal/apical/angular deviations	ARDN had higher angular deviation (3.72° vs. 3.1°, *p* = 0.02).
Obispo et al. (2023) [47]	AR appliance	Tooth preparation for crowns	Volumetric reduction, RMS alignment	AR improved precision (*p* = 0.0001) and conservatism.
Kihara et al. (2024) [48]	AR head-mounted display (HMD)	Tooth preparation	Over-reduction, angle accuracy	Cross-sectional AR reduced over-reduction (*p* < 0.05).
Lin et al. (2015) [49]	AR head-mounted display	Implant placement with surgical template	Entry/apex/angular/depth deviations	AR reduced deviations (entry: 0.50 mm, angle: 2.70°).
Pellegrino et al. (2019) [50]	HoloLens	Implant placement	Entry/apex/angular deviations	Feasibility confirmed (entry: 0.53 mm, angle: 3.05°).
Shusterman et al. (2024) [51]	ANNA^®^ (MR-DN system)	Implant placement	3D entry/apex deviations, angle	High accuracy (entry: 0.42 mm, angle: 1.85°).
AR in ITSP Education				
Daud et al. (2023) [52]	VR haptic simulator (unspecified)	Preclinical restorative training	Student perceptions, skill improvement	76% of students endorsed VR for supplemental training.
Mai et al. (2025) [53]	3D AR auto-evaluation algorithm	Tooth preparation evaluation	RMSE, time efficiency, user satisfaction	Reduced evaluation time (10.5 s vs. 2 h) (ICC = 0.75–0.95).
Grad et al. (2023) [54]	Microsoft HoloLens	Dental anatomy reconstruction	Hausdorff distance (Hmax)	3D-printed models outperformed AR (630 µm vs. AR, *p* = 0.004).
Özdemir et al. (2021) [55]	Virtual articulators	Occlusion analysis	Subjective usability	Enhanced dynamic occlusion teaching.
Li et al. (2021) [56]	VR simulators (multiple)	Preclinical skill training	Literature review	VR useful but limited by force feedback realism.
Mansoory et al. (2022) [57]	VR technology EKEN 4KUHD	Neutral zone/teeth arrangement	Test scores, student feedback	VR group scored higher (16.92 vs. 16.14, *p* < 0.05).
Alsufyani et al. (2023) [58]	VR panoramic anatomy software	Radiographic anatomy training	Landmark identification, satisfaction	Lecture-based outperformed VR (*p* < 0.005), but VR was engaging.
Arora et al. (2023) [59]	Virteasy haptic simulator	Crown preparation training	Preparation quality	Haptic simulators improved skills but conventional was better later (*p* < 0.05).
Hsu & Chang (2025) [60]	Simodont haptic simulator	Crown preparation prediction	Correlation with phantom head test	Simodont predicted success (OR = 5.6, *p* < 0.001).
Liebermann et al. (2024) [61]	Virtual prosthetic case planning app	Prosthetic case planning	Lecturer/student feedback	87% recommended integration into curricula.

## Data Availability

No new data were created or analyzed in this study. Data sharing is not applicable to this article.

## Supplementary Materials

The following supporting information can be downloaded at: https://www.mdpi.com/article/10.3390/dj13090435/s1, Supplementary File S1: Completed PRISMA-ScR Checklist; Supplementary File S2: Search strategy.

## Abbreviations

The following abbreviations are used in this manuscript:

AR	Augmented reality
ITSP	Implant and tooth-supported prosthodontics
PRISMA-ScR	Preferred Reporting Items for Systematic reviews and Meta-Analyses extension for Scoping Reviews
RCT	Randomized controlled trial
CAD/CAM	Computer-aided design/computer-aided manufacturing
3D	Three-dimensional
DSD	Digital smile design
AI	Artificial intelligence
MR	Mixed reality
MeSH	Medical Subject Headings
PICO	Patient/Problem, Intervention, Comparison, and Outcome.
PCC	Population, Concept, Context
JBI	Joanna Briggs Institute
