# Supplementary material for: Augmented Reality in Implant and Tooth-Supported Prosthodontics Practice and Education: A Scoping Review
Source: Dent J (Basel). 2025 Sep 21;13(9):435. doi: 10.3390/dj13090435 (PMC12468532; doi:10.3390/dj13090435)
Supplement: Supplementary file 1 [file dentistry-13-00435-s001.zip › Supplementary File S2 - Search.pdf]

Supplementary File S2

Database	Search strategy
PubMed accessed initially on February 12, 2025, then updated on July 25, 2025	(("augmented reality"[Title/Abstract] OR "mixed reality"[Title/Abstract] OR "AR"[Title/Abstract] OR "MR"[Title/Abstract] OR "virtual reality"[Title/Abstract] OR "VR"[Title/Abstract] OR "extended reality"[Title/Abstract] OR "XR"[Title/Abstract])<br>AND<br>("prosthodontics"[MeSH Terms] OR "prosthodontics"[Title/Abstract] OR "dental prostheses"[Title/Abstract] OR "dental implant"[Title/Abstract] OR "denture"[Title/Abstract] OR "crown"[Title/Abstract] OR "bridge"[Title/Abstract] OR "dental restoration"[Title/Abstract] OR "dental education"[Title/Abstract] OR "dental training"[Title/Abstract] OR "dental simulation"[Title/Abstract]))<br>NOT<br>("animals"[MeSH Terms] NOT "humans"[MeSH Terms])<br>AND<br>(("2015/01/01"[Date - Publication] : "2025/07/31"[Date - Publication]) AND English[Language])
Scopus accessed initially on February 11, 2025, then updated on July 25, 2025	TITLE-ABS-KEY (("augmented reality" OR "mixed reality" OR "AR" OR "MR" OR "virtual reality" OR "VR" OR "extended reality" OR "XR")<br>AND<br>("prosthodontics" OR "dental prostheses" OR "dental implant" OR "denture" OR "crown" OR "bridge" OR "dental restoration" OR "dental education" OR "dental training" OR "dental simulation"))<br>AND<br>(PUBYEAR > 2024 AND PUBYEAR < 2026)<br>AND<br>(LIMIT-TO (LANGUAGE , "English"))<br>AND<br>(EXCLUDE (SUBJAREA , "VETE") OR EXCLUDE (SUBJAREA , "AGRI"))
Web Of Science accessed initially on February 16, 2025, then updated on July 25, 2025	TS=(("augmented reality" OR "mixed reality" OR "AR" OR "MR" OR "virtual reality" OR "VR" OR "extended reality" OR "XR")<br>AND<br>("prosthodontics" OR "dental prostheses" OR "dental implant" OR "denture" OR "crown" OR "bridge" OR "dental restoration" OR "dental education" OR "dental training" OR "dental simulation"))<br>AND<br>PY=(2015-2026)<br>AND<br>LA=("English")<br>NOT<br>SU=("Veterinary Sciences" OR "Agriculture")

Embase accessed accessed initially on February 11, 2025, then updated on July 25, 2025	('augmented reality':ti,ab OR 'mixed reality':ti,ab OR 'ar':ti,ab OR 'mr':ti,ab OR 'virtual reality':ti,ab OR 'vr':ti,ab OR 'extended reality':ti,ab OR 'xr':ti,ab) AND ('prosthodontics':de OR 'prosthodontics':ti,ab OR 'dental prostheses':ti,ab OR 'dental implant':ti,ab OR 'denture':ti,ab OR 'crown':ti,ab OR 'bridge':ti,ab OR 'dental restoration':ti,ab OR 'dental education':ti,ab OR 'dental training':ti,ab OR 'dental simulation':ti,ab) AND [humans]/lim AND [2015-2026]/py AND [english]/lim
